# Immediate Compression of Common Illiac Artery

**Published:** 1874-04

**Authors:** Frank Woodbury


*AMERICAN JOURNAL OF THE MEDICAL SCIENCES—January,* 1874:

IMMEDIATE COMPRESSION OF THE COMMON ILIAC ARTERY.—*By Frank Woodbury, M.D.*—In the performance of an operation involving section of the femoral artery in its upper portion, the absolute control of the circulation through the vessel, for a few moments, is of inexpressible importance, and may decide the success or failure of the operation, the life or death of the patient.

In the examination of a case of nephritic colic, in which a stone was supposed to exist in the renal pelvis, or in the ureter, the writer found that while exploring the abdominal cavity, with the right hand and part of forearm in the rectum, he was able to observe that no stone of size existed in the parts indicated, and was struck by the novel sensation produced by the pulsation of the common iliac arteries and abdominal aorta immediately under the fingers. The idea occurred that the abdominal aorta and iliac vessels might be compressed and controlled with as much certainty and almost as readily as the radial artery. The value of this where the circulation is required to be checked for a brief period, in either or both lower extremities, is sufficiently evident, but is enhanced by the fact that the compression is made by a fleshy pad directly upon the vessel, and the venous trunk is avoided. The bowel should be evacuated by a large warm water injection previous to the operation. In controlling the right common iliac artery, the right hand of the assistant will be most convenient, and the left vessel will be most easily controlled by the left hand. In either case, the hand being anointed (with lard in preference to oil), and the fingers folded into a cone, it is gradually introduced into the rectum with its dorsum to the sacrum until reaching the sigmoid flexure, when the hand may be pronated, and, as the vessels are right under the fingers, the main supply of blood to the limb may thus, in a few moments, be completely controlled.

